# Prediction of Peak Overpressure of Charge Enveloped by Polymer Matrix Composite: Theoretical Modeling and Experimental Verification

**DOI:** 10.3390/polym15010219

**Published:** 2022-12-31

**Authors:** Jun-Bao Li, Wei-Bing Li, Xiao-Ming Wang, Jia-Xin Yu

**Affiliations:** ZNDY of Ministerial Key Laboratory, Nanjing University of Science and Technology, Nanjing 210094, China

**Keywords:** layered charge, peak overpressure, detonation products, prediction model, experimental verification

## Abstract

This study aimed at elucidating some characteristics of the shock wave overpressure generated by a non-traditional layered charge comprising an inner high-energy explosive and an outer polymer matrix composite. Two models for predicting the peak overpressure (Δ*p*_m_) of the charge were established, namely, a model based on the initial parameters of the blast wave, and a model considering the weakening of the explosion energy through the introduction of polymer matrix cladding. The overpressure of a typical layered charge was experimentally measured for model validation. It was found that the difference between the Δ*p*_m_ predicted by the two models and the experimental data is less than 15.12% and 14.17%, respectively. The model that was established based on the conservation of energy law, is in best agreement with the experimental data under different cladding/charge mass ratios (*α_m_*). The model that was based on the initial parameters of the blast wave obtained a low predicted value when *α_m_* was 0.4–0.8, which is attributed to the non-uniformity of the gas-solid mixture during the explosive dispersion stage.

## 1. Introduction

Cylindrical shells are widely used in traditional antipersonnel bombs, demolition bombs, and penetrating bombs. Many explosive charges used in modern warfare are designed as cylindrical rather than spherical forms due to the convenience of assembly. This research into a cylindrical layered charge with a non-metallic shell may provide guidance for the design of new-concept ammunition.

Recently, many studies have applied non-metallic and particle-filled non-metallic materials to blasting warhead projectiles to improve the damage efficiency of the warhead or enhance the designability of ammunition power [1,2,3,4]. For example, ammunition for low collateral damage is often fabricated using polymer matrix composites and a certain amount of explosives, then filled with heavy metal powders to produce different killing mechanisms [5,6]. The use of non-metallic materials, such as rubber and plastic matrix shells, is also considered based on certain performance requirements, including corrosion resistance and fatigue resistance [7,8,9]. A layered charge comprising an inner high-energy explosive and outer non-detonating material has gradually attracted research attention, owing to its different explosive energy output characteristics compared with a single charge. Studying the effect of non-detonating inert shells on the release of explosive energy from charges can extend the system for assessing the power of conventional blasting warheads, and provide guidance for the design of new weapons, such as underwater ammunition and tunable charges.

The introduction of non-detonating materials may result in the explosive reaction mechanism of the charge being significantly different from the energy output structure. On the one hand, the polymer matrix composite shell will undergo large deformation and dissipate energy in the early stages of the explosion, unlike traditional metal cylindrical shells [10,11]; on the other hand, the shell may disperse with the detonation products, which obviously affects the generation of a blast shock wave [12]. Considering the complexity of the dynamic mechanical properties and explosion response of non-detonation materials, many studies have investigated the explosive power of inert shell charges [13,14,15].

The fragmentation of glass-fiber-reinforced nylon shells driven by explosives has been experimentally investigated [13]. The velocity attenuation of non-metallic natural fragments was calculated by modifying the traditional resistance formula of spherical fragments. The characteristics of the underwater explosion shock wave of Trinitrotoluene (TNT) and H6 explosives enveloped by steel, polycarbonate plastic, and neoprene limb shells have been investigated via AUTODYN simulation [14]. It was found that the restraining effect of the shell is related to the shell’s material properties and detonation distance. Most of these studies conducted numerical simulation and experimental research, but in-depth analyses focusing on the theoretical aspects are lacking.

The theoretical evaluation of projectile explosion power is an important issue in the design of blasting warheads, and the calculation of shock wave overpressure is key for determining their energy output. Existing formulae for calculating the peak overpressure mainly involve the bare charge and the charge under the constraint of the metal shell. These formulae first determine the TNT equivalent of the charge and then calculate the corresponding peak overpressure at different positions from the charge center [16,17,18,19]. Regarding the calculation of shell charge peak overpressure, previous studies have mostly focused on the constraint conditions of thin-walled and high-strength metal shells, which mainly consume explosive energy through fracture [20,21].

Generally, the polymer matrix composite shell exhibits obvious energy absorption characteristics, which apparently affect the generation of an explosion shock wave. Consequently, the traditional method cannot be used to calculate the charge structure’s peak overpressure, and it becomes necessary to establish a theoretical model for predicting the value of the peak overpressure generated by a non-metallic shell charge. This study analyzed the mechanism of a charge enveloped by a polymer matrix composite shell. Two models are proposed to calculate the peak overpressure from the viewpoint of shock-wave generation and energy conservation during detonation and explosive dispersion. The proposed models were validated by experimentally measuring the shock wave for a charge enveloped by a filled rubber shell.

## 2. Mechanism of a Charge Enveloped by a Polymer Matrix Composite Shell

**Figure 1:**
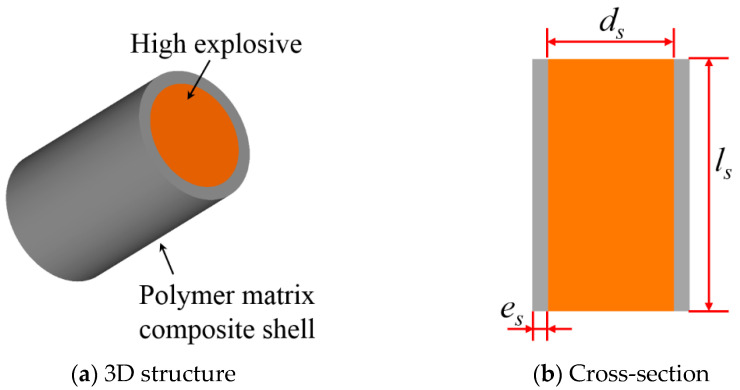
Structural diagram of a charge enveloped by a polymer matrix composite shell.

Figure 1 shows the schematic diagram of a typical layered charge, comprising an inner high-energy explosive and outer polymer matrix composite; *d*_s_ is the diameter of the inner explosive, *e*_s_ is the thickness of the outer layer, and *l*_s_ is the charge length.

After detonating high explosives, sufficient energy is generated to deform and break the shell. Therefore, the shell will be in a throwing state driven by the detonation products, and a series of physical interactions will occur in the high-temperature and high-pressure reaction space, resulting in an energy output difference compared with a conventional cylindrical charge.

**Figure 2:**
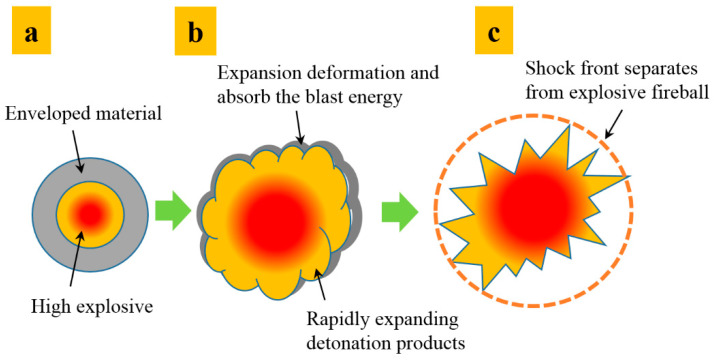
Explosion mechanism of charge enveloped by polymer matrix composite shell ((**a**–**c**) stages represent the detonation reaction of the inner charge, the mixing and expansion of the detonation products and cladding, the formation of the blast shock wave respectively).

Generally, the explosive reaction of a layered charge can be divided into the following three stages:

(1) The detonation reaction stage of the inner charge. As shown in Figure 2a, the explosive molecules undergo a rapid oxidation-reduction reaction after charge initiation, accompanied by the propagation of the detonation wave in the explosive. The detonation wave interacts with the cladding layer at the interface and causes shock-wave transmission and reflection.
(2) The mixing and expansion stages of the detonation products and cladding. As shown in Figure 2b, the detonation products expand without oxygen after detonation. Simultaneously, the cladding materials undergo considerable deformation during dispersion and absorb the explosion wave energy.
(3) The formation stage of the blast shock wave. As shown in Figure 2c, the mixture of the expanding detonation products and dispersed cladding will rapidly compress the air, leading to shock-wave formation in the air. Thereafter, the propagation speed of the primary shock wave will gradually become greater than the expansion speed of the detonation products, resulting in the shock wave separating from the fireball.

**Figure 3:**
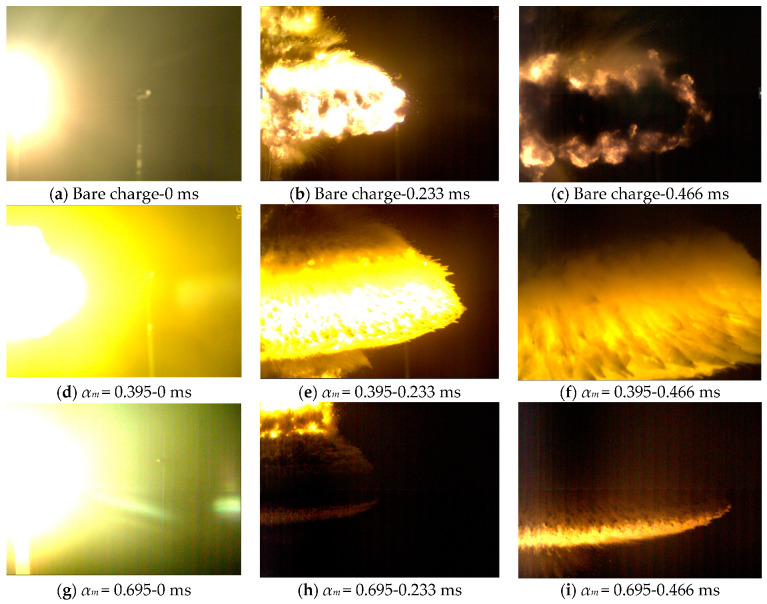
Typical explosion images of a charge enveloped by a non-metallic shell.

Figure 3 shows the explosion images of typical high explosives (JH-2 explosives) and charges with typical claddings of different masses (lithium fluoride/rubber composite), where *α_m_* refers to the mass ratio of cladding and explosive. As can be seen, the explosive fireball is approximately mushroom-shaped during expansion, and the introduction of cladding does not change the overall contour of the explosive fireball. When the *α_m_* value is too large, the dispersion speed of the detonation products is reduced, to a certain extent.

According to the mechanism of a charge enveloped by a non-metallic shell, the addition of the cladding layer affects the detonation product’s state parameters, such as the density, sound velocity, and particle velocity, thereby reducing the strength of the initial blast wave. On the other hand, the cladding absorbs and dissipates the energy of the detonation wave and explosion products during expansion, thereby weakening the blast wave at different distances from the detonation center. Therefore, it is necessary to calculate the peak overpressure of the layered charge, taking into account the initial parameters of the blast wave and energy conservation of explosive and cladding.

## 3. Prediction of Peak Overpressure Based on the Initial Parameters of the Blast Wave

### 3.1. Initial Blast Shock Wave Parameters

For a sparse medium such as air, the pressure on the initial shock wave front, *p*_x_, is far less than the detonation pressure, *p*_H_, of the explosive. The expansion of the detonation products from pressure *p*_H_ to *p*_x_ can be divided into two stages: the first stage is the expansion from *p*_H_ to boundary pressure *p*_k_, and the second stage is the expansion from *p*_k_ to the immediate pressure, *p*. Generally, the above-mentioned expansion process can be described by two adiabatic lines [16], as follows:

(1) $$\begin{array}{l} pv^{\gamma }=p_{H}v_{H}^{\gamma }\left(p_{k}\leq p\leq p_{H}\right)\\ pv^{k}=p_{k}v_{k}^{k}\left(p<p_{k}\right) \end{array}$$

where *p* and *v* are the pressure and specific volume of the explosive products; subscript H denotes the Chapman-Jouguet (C-J) detonation state [22]; subscript k is the boundary state of the two stages; *p*_k_ is expressed as follows:

(2) $$p_{k}=\frac{p_{H}}{2}\frac{k-1}{\gamma -k}{\left(\frac{\left(\gamma -1\right)Q_{v}}{\frac{1}{2}p_{H}v_{0}}-1\right)}^{\frac{\gamma }{\gamma -1}}$$

where *v*_0_ is the initial charge volume, and *Q*_v_ is the explosive heat of the charge, generally taken as 5.57 MJ/kg [22]; *v*_k_ is the volume at the boundary state.

This study recalculated the particle velocity of the gas-solid mixture, consisting of detonation products and dispersed cladding at the boundary point, based on the assumption that the cladding does not change the expansion of the detonation products of the inner charge. For the second stage, the density and sound velocity of the gas-solid mixture are calculated, and then the initial parameters of the layered-charge shock wave are determined.

When the explosion product expands from *p*_H_ to *p*_k_, the product velocity increases from *u*_H_ to *u*_k_, as follows:

(3) $$u_{k}=u_{H}+\int_{p_{k}}^{p_{H}}\frac{v}{C}dP.$$

For a gas-solid mixture comprising detonation products and dispersed cladding, the particle velocity can be expressed as follows [17]:

(4) $$u_{e}=\sum_{i=1}^{n}a_{i}u_{i}^{2}$$

where *a*_i_ and *u*_i_ are the specific gravity and particle velocity of the *i*^th^ phase.

During the expansion of the explosion products from *p*_k_ to the initial pressure *p*_x_ of the shock wave, an isentropic assumption can be made because the pressure is relatively low. The sound velocity, *C*, is defined as follows:

(5) $$C={\sqrt{\left(\frac{\partial p_{m}}{\partial \rho_{m}}\right)}}_{S}$$

where *p*_m_ and *ρ*_m_ are the pressure and density, and the following relationship holds:

(6) $$\frac{C_{x}}{C_{k}}={\left(\frac{v_{x}}{v_{k}}\right)}^{-\frac{k-1}{2}}={\left(\frac{p_{x}}{p_{k}}\right)}^{\frac{k-1}{2k}}$$

where subscript x represents the state of detonation products when the initial shock wave is formed. The sound velocity of the gas-solid mixture can be further calculated, as follows [18]:

(7) $$\frac{a_{0}}{a_{e}}=\frac{1}{1+x\beta }\sqrt{\frac{\gamma \left(1+\delta \beta \right)\left(1+\beta \right)}{\gamma +\delta \beta }}$$

where *a*_0_ is the gas phase sound velocity, *x* is the gas-solid density ratio, *β* is the solid-gas mass ratio, *γ* is the gas adiabatic index, generally taken as 1.4 [22], and *δ* is the solid/gas specific heat ratio. The density of the gas-solid mixture can be determined as follows:

(8) $$\frac{m}{\rho_{e}}=\sum_{i=1}^{n}\frac{m_{i}}{\rho_{i}}$$

where *m* and *m_i_* are the mass of the mixture and the *i*^th^ phase. When the product pressure expands from *p*_k_ to *p*_x_, the product speed will increase from *u*_e_ to *u*_x_, as follows:

(9) $$u_{x}=u_{e}+\int_{p_{x}}^{p_{k}}\frac{v}{C}dP=u_{e}+\int_{p_{x}}^{p_{k}}\frac{v}{C}dP$$

Considering that the initial explosion shock wave is a strong shock wave, the following relationship holds [16]:

(10) $$p_{x}=\frac{K_{a}+1}{2}\rho_{a}u_{x}^{2}$$

where *K*_a_ and *ρ*_a_ are the isentropic index and density of undisturbed air, respectively. Finally, the initial parameters of the explosion shock wave can be determined by combining Equations (2) and (10). The calculation results obtained from several *α_m_* values are presented in Table 1.

### 3.2. Scaling Law of Explosion

According to the scaling law of explosion in an infinite air medium, the typical shock wave parameters, namely, the peak overpressure, Δ*p*_m_, positive pressure duration, *t*_+_, and specific impulse, *i*_I_, can be obtained based on dimensional analysis and test calibration; Δ*p*_m_ is expressed as follows:

(11) $$\Delta p_{m}=a_{0}+\frac{a_{1}}{\bar{R}}+\frac{a_{2}}{{\bar{R}}^{2}}+\frac{a_{3}}{{\bar{R}}^{3}}+\ldots$$

where $\bar{R}=R/\sqrt[{3}]{W_{c}}$ and *W*_c_ is the charge mass, *R* is the distance from the explosion center, *a_i_*, *b_i_*, and *c_i_* are constants, and *i* = 0, 1, 2, 3….

Based on a large amount of test data, the formula for calculating the peak overpressure of a spherical TNT explosion in infinite air is expressed as follows [16]:

(12) $$\Delta p_{m}=0.082\frac{1}{\bar{R}}+0.265\frac{1}{{\bar{R}}^{2}}+0.686\frac{1}{{\bar{R}}^{3}},\;1\leq \bar{R}\leq 15$$

Generally, when the shock wave propagation distance is greater than the charge length, a cylindrical charge can be directly considered as a spherical charge with the same mass.

**Figure 4:**
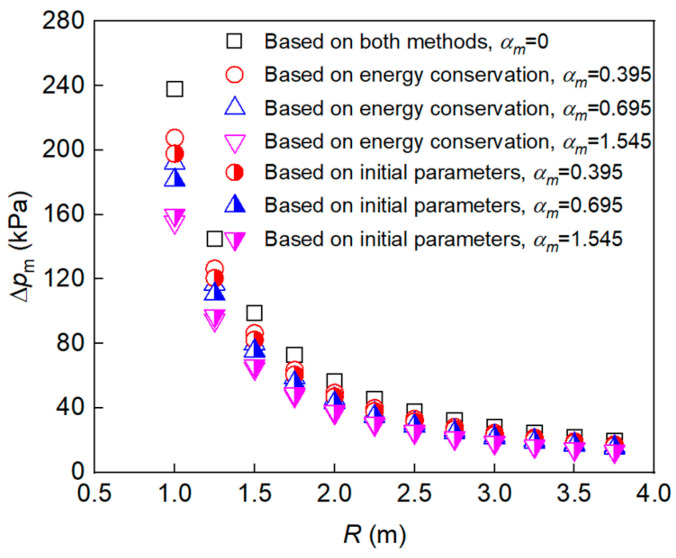
Calculations of peak overpressure for charges with different distances (the prediction results of the two methods are consistent at *α*_m_= 0).

**Figure 5:**
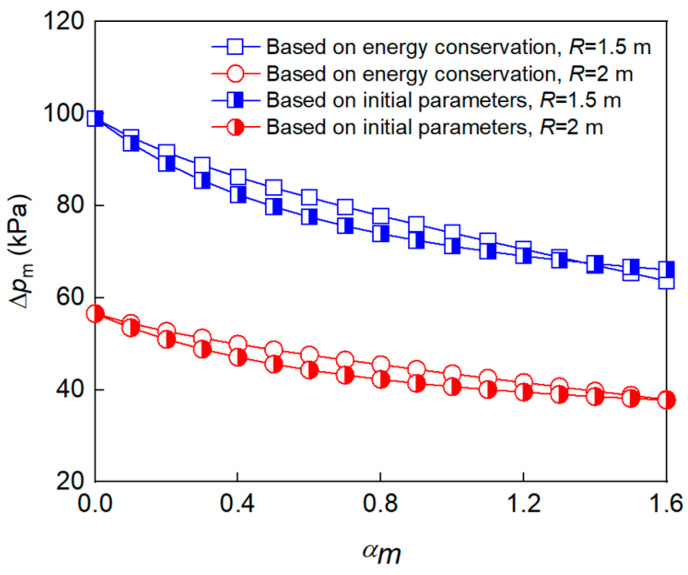
Calculations of peak overpressure for charges with different mass ratio.

To determine the value of overpressure, the initial outflow position of the shock wave was determined by Equations (1) and (2), and the initial pressure, px, was calculated using Equation (10). Combined with the initial pressure, outflow position, and Equation (12), the equivalent charge mass was calculated and the overpressure at different propagation distances was obtained. The relationships between the peak overpressure and distance and the cladding/charge mass ratio were calculated, as shown in Figure 4 and Figure 5. As can be seen, the trend of the peak overpressure with the propagation distance is consistent with that of the bare charge. As the distance from the explosion center increases, the influence of the inert cladding mass on the peak overpressure becomes smaller. It should be noted that the excessive mass ratio of a non-metallic shell will significantly reduce the overpressure of the charge, which is obviously not in line with reality in the application scenario. Therefore, this paper referred to the design criteria of conventional blasting warheads and determined an appropriate loading ratio range of 0.4–1; that is, the range of the mass ratio is about 0–1.5.

## 4. Prediction of Peak Overpressure Based on the Conservation of Energy Law

Based on the initial parameters of the blast wave, the model for calculating the peak overpressure of layered charge was established as described above. Considering the conservation of energy of the explosive and the cladding, there is another way to calculate the peak overpressure. When solving the explosion shock wave of different charge types in an infinite air medium, it is necessary to determine the TNT equivalent, *W*_T_, as follows:

(13) $$W_{T}=\frac{Q_{\mathrm{vi}}}{Q_{\mathrm{vT}}}W_{i}$$

where *Q*_vi_ and *W*_i_ are the detonation heat and mass of the charge, respectively, and *Q*_vT_ is the detonation heat of TNT.

Unlike the problem of the explosion of a bare explosive, the blast wave of a layered charge is more complex and tends to be affected by the inherent physical and chemical properties of the outer cladding and the deformation and movement occurring during explosion dispersion. On the one hand, shell deformation and fragmentation consume the energy released by explosives; on the other hand, the cladding affects the expansion of detonation products and the subsequent shock-wave formation. The effect of traditional metal shells on the energy output of charges is reflected by the transformation of explosive energy to shell kinetic energy. It was found that the deformation and crushing energy of cylindrical metal shells are often less than 3% of the energy released by the explosive [16]. Owing to the strong cushioning ability of the polymer matrix composite, the non-metallic shell exhibits different mechanisms of influence on the layered-charge shock wave.

For charges with cladding, the total energy output, *E*, can be expressed as follows:

(14) $$E=E_{a}+E_{k}+E_{s}$$

where *E*_a_ is the shock wave energy absorbed and dissipated by the cladding, *E*_k_ is the kinetic energy of the dispersed cladding, and *E*_s_ is the energy driving the detonation products to expand and generate the initial shock wave.

### 4.1. Energy of the Detonation Products

The energy of detonation products consists of the internal energy, *E*_1_, and kinetic energy, *E*_2_. Generally, it is considered that the pressure *p* of the detonation products during expansion is expressed as follows:

(15) $$p=A_{d}\rho_{d}^{\gamma_{d}}$$

where *A*_d_ is a constant, generally taken as 2 [23], *ρ*_d_ is the density of the detonation products, and *γ*_d_ is the polytropic exponent. The internal energy can be expressed as follows [16]:

(16) $$E_{1}=\frac{m_{0}p_{d}v_{d}}{\gamma_{d}-1}$$

where *v*_d_ is the volume of the detonation products. Assuming that the internal pressure distribution of the detonation products is uniform, the kinetic energy of the products can be expressed as follows:

(17) $$E_{2}=\frac{m_{0}}{2\left(a+1\right)}u_{k}^{2}$$

where *u*_k_ is the expansion velocity of the shell, and *a* is the shape coefficient, generally taken as 1.

### 4.2. Energy of the Cladding

The energy conversion involved in the explosion process of the cladding material mainly includes the enhancement of the cladding’s internal and kinetic energy.

When the explosion wave propagates to the surrounding medium, its energy can be converted into the irrecoverable internal energy of the medium, and the energy is dissipated due to material failure. According to the relationship of the shock-wave front, the change of the material’s internal energy on the wavefront can be expressed as follows [19]:

(18) $$E_{a}=E_{0s}-E_{0}=\frac{1}{2}\left(p_{0s}+p_{0}\right)\left(v_{0}-v_{0s}\right)$$

Compared with *p*_0s_, *p*_0_ is generally negligible; therefore, Equation (18) can be written as follows:

(19) $$E_{a}=\frac{1}{2}p_{0s}\left(v_{0}-v_{0s}\right).$$

The energy dissipation mechanism of multi-component composites is more complex, and the detonation energy consumed in the expansion deformation under high detonation pressure cannot be ignored. For polymer matrix composites, the work done by the materials under impact compression has obvious irreversible characteristics, causing the isentropic unloading line [19] to be far lower than the impact adiabatic line. Additionally, the unloading of the shock wave energy of the polymer matrix composites is often lower than that of metals, owing to its inherent viscosity. Consequently, the material retains a considerable part of the internal energy during expansion with the detonation products, which obviously does not contribute to the initial shock-wave generation. Therefore, this study ignored the contribution of the internal energy enhancement of the cladding after shock compression to the formation of the layered charge’s shock wave.

For the layered charge, the increase in the cladding’s specific internal energy can be calculated, based on the initial pressure at the explosive-cladding interface and the *p-v* relationship of the material. When the detonation wave reaches the shell’s inner surface, a reflected wave is generated, followed by pressure, *p*_r_, and density, *ρ*_r_, which obey the following relationship [19]:

(20) $$p_{r}=p_{C-J}{\left(\frac{\rho_{r}}{\rho_{C-J}}\right)}^{\gamma_{d}}$$

where subscript C-J represents the stable detonation state. Here, the detonation parameters of the explosive were obtained from the literature [20].

When the reflected wave is a shock wave, the relationship between the pressure, *p*_r_, and particle velocity, *u*_r_, following the reflected wave can be expressed as follows:

(21) $$u_{r}-u_{C-J}=\sqrt{\left(p_{r}-p_{C-J}\right)\left(\frac{1}{\rho_{C-J}}-\frac{1}{\rho_{r}}\right)}$$

where *u*_C-J_ is the particle velocity of the detonation products.

When the reflected wave is a rarefaction wave, according to the relationship of one-dimensional isentropic motion, the eigenvalue before and after the reflected wave remains unchanged, and the following relationship is obtained [17]:

(22) $$u_{r}-u_{C-J}=-\int_{p_{C-J}}^{p_{r}}\frac{dp}{\rho C}.$$

By combining the definition of sound velocity and the state equation of the detonation products, the *p*_r_-*u*_r_ relationship can be obtained, as follows:

(23) $$u_{r}-u_{C-J}=\frac{2}{K-1}\sqrt{\frac{Kp_{C-J}}{\rho_{C-J}}}\left[1-{\left(\frac{p_{r}}{p_{C-J}}\right)}^{\frac{K-1}{2K}}\right].$$

**Figure 6:**
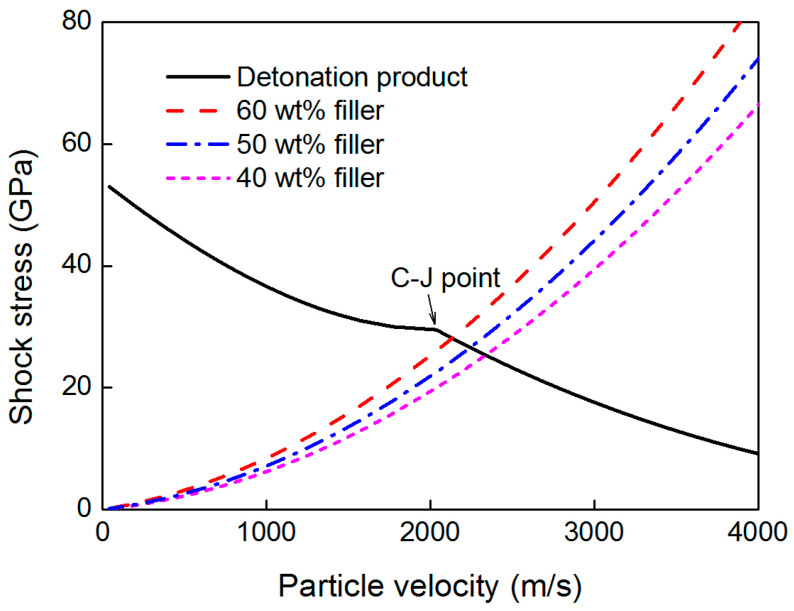
Relationship between pressure and particle velocity for detonation products and cladding materials.

The *p-u* relationship of the reflected wave of the detonation products can be calculated from Equations (21) and (23). Figure 6 shows the calculated *p-u* curve of the detonation products of the JH-2 explosive, and three formulations of the lithium fluoride-filled rubber [9]. The explosive’s C-J point can be considered as the dividing point; the upper part indicates that the reflected wave is a shock wave, while the lower part indicates that the reflected wave is a rarefaction wave. Then, the initial pressure at the interface can be obtained.

The detonation wave generated by the inner charge is considered to act on the explosive-cladding interface at a certain angle. The angle *α* along the detonation wave propagation direction gradually increases from 0 to arctan (2*l*/*d*). Hence, coefficient *θ* is introduced to calculate the initial pressure, *p*_i0_′, as follows:

(24) $$p_{i0}\prime =\theta p_{i0}$$

(25) $$\theta =\frac{1+\sin\left(\alpha \right)}{2}$$

For a cylindrical charge, the cylinder’s steady expansion velocity can be determined using the Gurney formula, as follows [21]:

(26) $$u_{k}=\sqrt{2E_{g}}{\left(\frac{m_{g}}{m_{0}}+\frac{1}{2}\right)}^{-\frac{1}{2}}$$

where $\sqrt{2E_{g}}$ is the Gurney velocity, which can be calculated according to the adiabatic index and density of the explosive; *m*_g_ is the shell mass; *m*_0_ is the explosive mass. For a shell made of filled composite, the phase gaps in the material lead to the outflow of detonation products and energy loss during fracture and explosion dispersion. Therefore, a coefficient, *μ*, considering the loss of explosive product is introduced, and the stable expansion velocity is expressed as follows:

(27) $$u_{k}=\mu \sqrt{2E_{g}}{\left(\frac{m_{g}}{m_{0}}+\frac{1}{2}\right)}^{-\frac{1}{2}}$$

Based on a large number of experimental data, Chen et al. [22] reported that *μ* = 0.75 is acceptable; therefore, the kinetic energy of the cladding can be expressed as follows:

(28) $$E_{p}=0.5625E_{g}m_{g}{\left(\frac{m_{g}}{m_{0}}+\frac{1}{2}\right)}^{-1}$$

To determine the value of overpressure, the explosive equivalent used for shock-wave generation was first calculated using Equations (14), (19) and (28). Then, the TNT explosive equivalent for shock-wave generation was further obtained by using Equation (13). Finally, the value of overpressure at different propagation distances was obtained using Equation (12), as shown in Figure 4 and Figure 5. As can be seen, the peak overpressure at 1 m decreases by up to 41.7% when αm is 1.545.

## 5. Experimental Verification

### 5.1. Experimental Design

A shock-wave overpressure test on a layered charge that is enveloped by a typical filled polymer material was conducted to validate the established peak overpressure model.

The layered charge used in the experiments included an internal JH-2 explosive, with a diameter of 35 mm, and a non-metallic shell. The height of the charge was 80 mm. The JH-2 explosive was composed of 95 wt % cyclo-1,3,5-trimethylene-2,4,6-trinitramine (RDX), 3 wt % C7H6N2O4 (DNT), and 2 wt % polyvinyl acetate (CZ) with a density of 1.70 g/cm^3^ pressed with a pressing machine.

The non-metallic shell was lithium fluoride powder/rubber (LiF/NR) composites (Shandong Non-Metallic Materials Institute, China). When processing this composite, the rubber was first refined on an XK-400 open mixing mill. The LiF powder, with an average diameter of 50 μm, was then added, and the volume ratios of fillers to the rubber master batch were set to 5:5. After that, the rubber was evenly kneaded at the minimum roller distance and thinned by passing it through the roller 4–5 times. Finally, the refined rubber was vulcanized and molded by a 200 T vulcanizer [4]. The density, *ρ*_shell_, and flow stress, *σ*_0.2_, as well as the Hugoniot parameter, *c*_0_ and *λ*, of the composite are provided in Table 2. Three shells with different thicknesses, *d*_shell_ (3 mm, 5 mm, and 10 mm), were used; the corresponding *α_m_* values are listed in Table 3.

**Figure 7:**
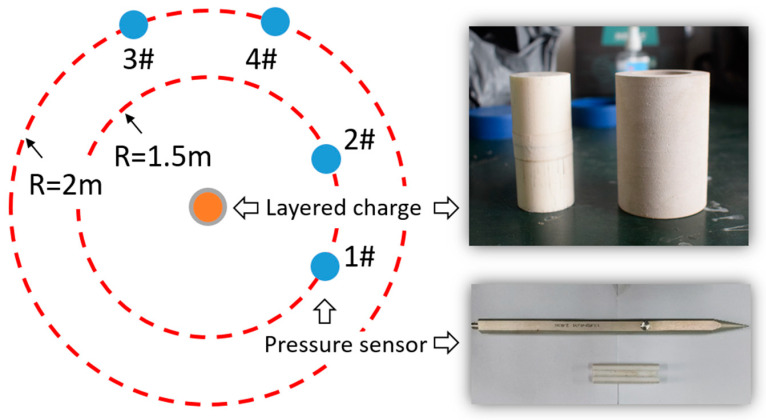
Details of the test site layout.

From the test device in Figure 7, it can be seen that a support with a height of 1.46 m was placed on the ground. Two free-field pressure sensors (Y3003, Yangzhou Ryan Electronic Technology Co., Ltd., Yangzhou, China) were placed at distances of 1.5 m and 2 m from the explosion center and then installed on a metal bracket at a height of 1.5 m from the ground, to ensure that the sensitive surface and charge center were at the same height. In the test, the initiation was realized with a #8 electric detonator. An initial explosion shock wave was generated during the expansion of the detonation product. Then, the wavefront passed over the location of the overpressure sensor and gradually decreased with the increasing propagation distance.

### 5.2. Model Verification

**Figure 8:**
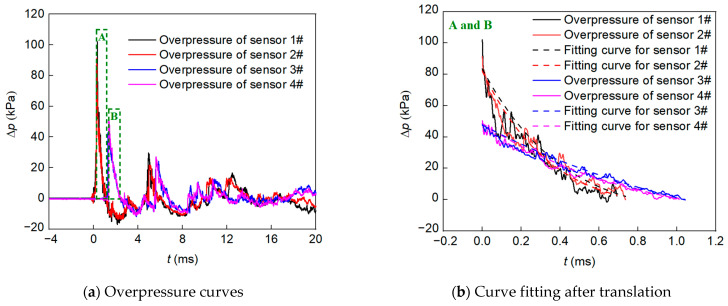
Typical overpressure curves and the determination of peak overpressure.

Figure 8 shows the typical overpressure curves of test No. 003 at 1.5 m and 2 m from the charge center. As can be seen, the pressure curves at the same distance are very similar. As the distance to the charge center decreases, the pressure decreases faster after reaching the peak value, and the negative pressure area becomes larger afterward. Considering the overpressure curve fluctuation, the following formula is used to determine the peak value of overpressure [5]:

(29) $$\Delta p=\Delta p_{m}e^{-\chi t}(1-\frac{t}{t_{+}})$$

where *χ* is the attenuation coefficient. Zones A and B in Figure 8a are nonlinearly fitted (Figure 8b). The curve-fitting parameters for overpressure are provided in Table 4. As can be seen, Δ*p*_m_ decreases while *t*_+_ increases with distance, which is related to the attenuation effect of the shock wave in the air.

**Figure 9:**
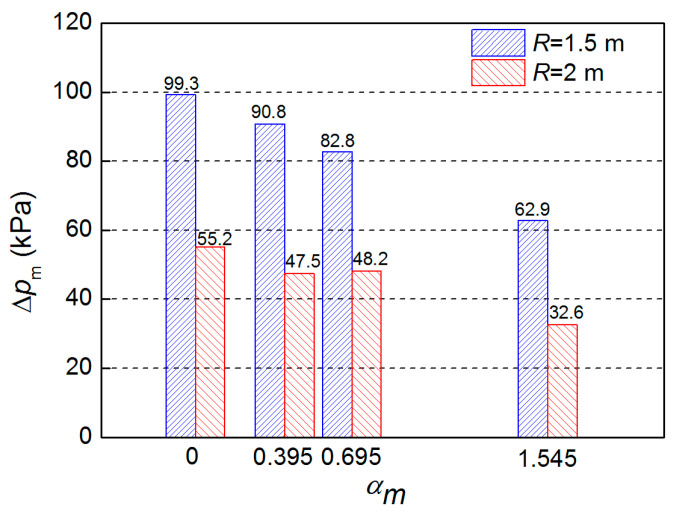
Peak overpressure under different *α_m_* values.

**Figure 10:**
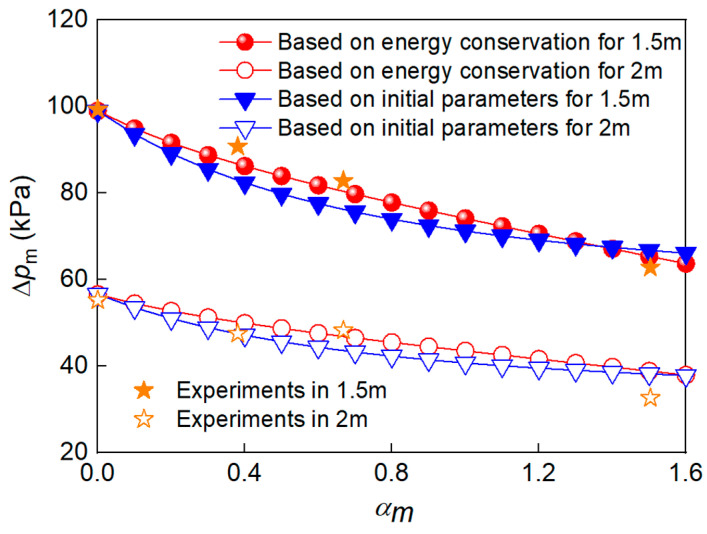
Comparison between Δ*p*_m_ values for different *α_m_* values in the theoretical calculation and experimental measurement.

Figure 9 shows the peak overpressure at different *α_m_* values. When *α_m_* increased from 0.395 to 1.545, Δ*p*_m_ at 1.5 m and 2 m from the charge center decreased by 30.8% and 31.3%, respectively. By comparing the peak overpressure in Figure 10 and Table 5, it can be seen that the theoretical calculation and test results are in good agreement, and the prediction error for the two models is less than 15.12% and 14.17%, respectively. The model based on energy conservation better describes the trend of Δ*p*_m_, while the model based on continuous conditions obtained slightly smaller prediction values at the *α_m_* range of 0.4–0.8, which is attributed to the non-uniformity of the gas-solid mixture during the second expansion stage. It should be noted that the peak overpressure for α_m_ = 0.695 in 2 m is slightly lower than that for α_m_ = 0.395, which may be related to the non-ideal factors, such as the presence of the sensor support, the slight inclination of the sensitive surface, and the unevenness of the ground.

## 6. Conclusions

This study investigated the shock wave overpressure generated by a layered charge consisting of an inner high-energy explosive and an outer polymer matrix composite. Two models for predicting the peak overpressure (Δ*p*_m_) of the layered charge were established, based on an equation of state calculation for the mixture of detonation products and dispersed cladding and the energy conservation of the explosive and cladding. The difference in the obtained Δ*p*_m_ in relation to the model prediction and experimental measurements is less than 15.12% and 14.17%, respectively. The established model considering the weakening of the explosion energy via the introduction of a non-detonating layer is in best agreement with the experimental data under a cladding/charge mass ratio range of 0–1.545. The model based on the initial parameters of the blast wave obtained a low predicted value when *α_m_* was in the range of 0.4–0.8, which can be attributed to the non-uniformity of the gas-solid mixture during the explosive dispersion stage.

## Figures and Tables

**Table 1:** Detailed parameters of the blast shock wave for different *α_m_* values.

No.	*α_m_*	*p*_x_ (MPa)	*u*_x_ (m/s)	*c*_k_ (m/s)
001	0	87.5	7478	905
002	0.395	72.8	7036	1136
003	0.695	66.8	6740	1282
004	1.545	58.7	6321	1621

**Table 2:** The characteristics of the investigated LiF/NR composite.

Material	*ρ*_shell_ (g/cm^3^)	*σ*_0.2_ (MPa)	*c*_0_ (m/s)	*λ*
LiF/NR composite	1.82 ± 0.02	2.945	2210	1.96

**Table 3:** Experimental schemes and mass ratio of the charge for the tests.

Test No.	*d*_shell_ (mm)	*m*_0_ (g)	*m*_g_ (g)	*α*_m_
001	0	65.42	0	0
002	3	65.39	25.83	0.395
003	5	65.42	45.47	0.695
004	10	65.40	101.04	1.545

**Table 4:** The curve-fitting parameters for overpressure of test No. 003.

Sensor	Δ*p*_m_ (kPa)	*χ*	*r* ^2^
1#	82.21	1.549	0.944
2#	83.45	1.930	0.928
3#	48.75	0.725	0.983
4#	47.66	0.402	0.981

**Table 5:** Comparison between the theoretical calculation and experimental measurement of peak overpressure under different *α_m_* [4].

Test No.	1.5 m					2 m				
	Exp. (kPa)	Theory (kPa)	Error (%)	Theory (kPa)	Error (%)	Exp. (kPa)	Theory (kPa)	Error (%)	Theory (kPa)	Error (%)
001	99.3	99	0.30	99	0.30	55.2	56.6	−2.47	56.6	−2.47
002	90.8	86.4	5.15	82.5	10.02	47.5	50.0	−4.96	47.2	0.68
003	82.8	79.8	3.70	75.7	9.38	48.2	46.5	3.56	43.3	11.38
004	62.9	64.6	−2.64	66.5	−5.35	32.6	38.4	−15.1	38.0	−14.2

## Data Availability

The data presented in this study are available on request from the corresponding author upon reasonable request.
